# Mortality after chronic subdural hematoma is associated with frailty

**DOI:** 10.1007/s00701-022-05373-w

**Published:** 2022-09-29

**Authors:** Jurre Blaauw, Bram Jacobs, Heleen M. den Hertog, Niels A. van der Gaag, Korné Jellema, Ruben Dammers, Kuan H. Kho, Rob J. M. Groen, Joukje van der Naalt, Hester F. Lingsma

**Affiliations:** 1 Department of Neurology, University of Groningen, University Medical Center Groningen, Groningen, The Netherlands; 2 Center for Medical Decision Sciences, Department of Public Health, Erasmus Medical Center, Rotterdam, The Netherlands; 3 Department of Neurology, Isala Hospital Zwolle, Zwolle, The Netherlands; 4 University Neurosurgical Center Holland (UNCH), Leiden University Medical Center, Haaglanden Medical Center & Haga Teaching Hospital, Leiden & The Hague, The Netherlands; 5 Department of Neurology, Haaglanden Medical Centre, The Hague, The Netherlands; 6 Department of Neurosurgery, Erasmus MC Stroke Center, Erasmus Medical Center, Rotterdam, The Netherlands; 7 Department of Neurosurgery, Medisch Spectrum Twente, Enschede, The Netherlands; 8 Clinical Neurophysiology Group, Faculty of Science and Technology, University of Twente, Enschede, the Netherlands; 9 Department of Neurosurgery, University Medical Center Groningen, Groningen, The Netherlands

**Keywords:** Chronic Subdural hematoma, Frailty, Mortality, Survival

## Abstract

**Purpose:**

Chronic subdural hematoma (CSDH) is a common neurological disease often affecting the elderly. Long-term excess mortality for patients after CSDH has been suggested but causes of death are unknown. We hypothesize that excess mortality of CSDH patients is related to frailty. In this article, we describe mortality rates and causes of death of CSDH patients compared with the general population and assess the association of frailty with mortality.

**Methods:**

A cohort study in which consecutive CSDH patients were compared to the general population regarding mortality rates. Furthermore, the association of six frailty indicators (cognitive problems, frequent falling, unable to live independently, unable to perform daily self-care, use of benzodiazepines or psychotropic drugs, and number of medications) with mortality was assessed.

**Results:**

A total of 1307 CSDH patients were included, with a mean age of 73.7 (*SD* ± 11.4) years and 958 (73%) were male. Median follow-up was 56 months (range: 0–213). Compared with controls CSDH patients had a hazard ratio for mortality of 1.34 (95% CI: 1.2–1.5). CSDH patients more often died from cardiovascular diseases (37% vs. 30%) and falls (7.2% vs. 3.7%). Among CSDH patients frequent falling (HR 1.3; 95% CI: 1.0–1.7), inability to live independently (HR 1.4, 95% CI: 1.1–1.8), inability to perform daily self-care (HR 1.5; 95% CI: 1.1–1.9), and number of medications used (HR 1.0; 95% CI: 1.0–1.1) were independently associated with mortality.

**Conclusions:**

CSDH patients have higher mortality rates than the general population. Frailty in CSDH patients is associated with higher mortality risk. More attention for the frailty of CSDH patients is warranted.

## Introduction

Chronic subdural hematoma (CSDH) is a frequently occurring neurological disease that often requires neurosurgical intervention. CSDH is especially common in the elderly with incidences rapidly increasing with age [20]. Whereas CSDH has been considered as a benign disease in the past, reported twelve-month mortality is around 15–20% and can be as high as 32% [22, 25, 29]. This mortality rate is comparable with certain cancer types, such as colon carcinoma, larynx carcinoma, and non-Hodgkin lymphoma [21]. Furthermore, several studies have reported longer-term excess risk of mortality in CSDH patients compared with the general population, demonstrating the large impact of CSDH on survival [29]. It has been proposed that CSDH should therefore be considered as a sentinel event in the elderly, comparable with hip fractures [12, 25].

Several explanations for the increased mortality of CSDH patients have been suggested, including the use of specific medication, polypharmacy, presence of comorbidity, cerebral atrophy or underlying chronic disease [22, 25, 29]. Most of these risk factors are also related to advanced age and can be part of the “frailty syndrome”, more commonly referred to as frailty [7, 8, 40]. Frailty does not have a standard definition but is often regarded as a deterioration of multiple systems such as the neurological, musculoskeletal, cardiovascular, metabolic, and/or immunological systems [15, 37]. Another point of view on frailty is the loss of physiological reserves and loss of the ability to overcome a disease or injury leading to vulnerability for an adverse health outcome [9]. Frailty can be measured with different scales, mostly containing information on mobility, strength, medication use, comorbidity, and cognitive status [9–11].

Studies regarding frailty in CSDH mostly focus on predicting surgical outcome or 30-day functional outcomes [19, 32, 33]. In contrast to a number of other conditions, no studies are available which address the effect of frailty on the excess mortality of CSDH patients [3, 13, 27]. In previous studies, it has been shown that frailty can be improved, which leads to lower mortality rates [35, 37]. Therefore, determining whether excess mortality of CSDH patients is related to frailty could be an important first step in the process of reducing this mortality.

The aim of this study is to describe long-term mortality rates and cause of death of CSDH patients in comparison with the general population. Second, we assessed the association of frailty with mortality with the hypothesis that mortality in CSDH patients is associated with frailty.

## Methods

### Study population

All patients with CSDH who presented in three hospitals in the Netherlands between 2005 and 2019 were retrospectively included. The including hospitals were the University Medical Center Groningen, Isala Hospitals Zwolle, and Medisch Spectrum Twente Enschede. Inclusion periods varied from 8 to 13 years. The three institutions provide neurosurgical facilities and serve as referral centers for all patients requiring neurosurgical treatment in their region.

Patients were eligible if they were 18 years or older. CSDH was defined as a hypodense hematoma with a maximum of 1/3 of hyperdense components. Patients with a history of cerebral vascular malformation or intracranial tumor were excluded, as the required surgical intervention in these cases could lead to the development of a CSDH.

### Controls

We used data of the Dutch Central Bureau of Statistics (CBS), a governmental organization that collects and analyses (among others) mortality data in the Netherlands for epidemiological purposes. From the anonymized CBS database, we created a control group by selecting four controls for each CSDH patient, matched for sex and month and year of birth, by selecting four controls with the same sex with date of births closest to date of births of the CSDH patient. To be eligible for the matched control group, controls had to be alive at the day their matched CSDH patient was diagnosed with CSDH.

### Measures and definitions

Patient demographics included age and sex, Glasgow Coma Scale (GCS) score, and comorbidity measured by the Charlson Comorbidity Index (CCI). The CCI is a score that categorizes comorbidity based on the International Statistical Classification of Diseases and Related Health Problems (ICD). In the CCI, points are given based on the presence or absence of conditions, resulting in a total score that relates to survival [5, 6].

Treatment modality (surgery yes or no), side of hematoma, and millimeters midline shift (based on CT scan) were collected, as well as the Markwalder grading scale (MGS) and modified Rankin (mRS) score at diagnosis [4, 23, 28]. The MGS is a clinical grading scale ranging from 0 to 4 specifically designed for CSDH. An MGS score of 0 indicates no complaints and a score of 4 is for stuporous non-responding patients (GCS score ≤ 8). The mRS reflects functional outcome ranging from 0 (no symptoms) to 6 (death). All data were retrieved from the electronic patient files.

### Ethical consent

The local medical ethical committees of the participating hospitals approved this study and due to the retrospective nature of the study, the need for informed consent was waived.

### Frailty indicator

Frailty was determined using six different items, which were scored to be present or absent at the time of diagnosis:

1. Cognitive problems, either self-reported by patient/next of kin or diagnosed by the treating physician
2. Frequent falling, defined as three times or more in the last twelve months, either self-reported or resulting in a recorded visit to the emergency department
3. Unable to live independently, defined as need for home or informal care, (assisted) living, or living in a nursing home/rehabilitation center
4. Unable to perform daily self-care, expressed by a modified Rankin scale score of 4 or more
5. Usage of benzodiazepines or psychotropic drugs (e.g., clozapine, haloperidol)
6. Total number of medications used.

### Outcome

The primary outcome of this study was survival, based on data of the Dutch Central Bureau of Statistics (CBS). If patients or controls were registered as deceased, date and cause of death categorized by ICD-10 was collected.

### Statistical analyses

We calculated median survival time (median time from diagnosis until death in all patients that were deceased) and median follow-up time (median time from diagnosis until last known time point for all patients).

We used Kaplan–Meier curves with log-rank tests and Cox proportional hazard analysis to compare the mortality rates of CSDH patients and the matched controls. Mann–Whitney *U* tests were used to compare causes of death of CSDH patients and matched controls.

Within the cohort of CSDH patients, chi-square or Mann–Whitney *U* tests were used to compare baseline characteristics and frailty for CSDH patients who were alive vs. those who had died at the median survival time. We used Cox proportional hazard analysis to assess the association between the elements of the frailty indicators and survival, adjusted for age, sex, CCI score, treatment modality, and MGS score at admission. Furthermore, we assessed the relation between survival and the following factors potentially associated with mortality; age, sex, CCI score, treatment modality, MGS score at admission, and midline shift. The associations were presented as hazard ratios (HR) with a corresponding 95% confidence interval (CI).

All statistical analyses were performed with IBM SPSS 25. Figures were made using the “survival,” “survminer,” and “ggplot” packages in R. Patients with missing baseline data were excluded from analyses for those specific variables.

## Results

A total of 1307 CSDH patients were included. Mean age was 73.7 (*SD* ± 11.4) years and 958 (73%) were male (Table 1). The majority of patients received surgical treatment of which 838 (64%) received only surgery. Eighty patients (6%) received dexamethasone treatment, 191 (15%) patients received both dexamethasone and surgery, and a wait-and-see policy was chosen in 197 (15%) of patients. Unilateral hematoma was present in 955 (73%) patients with a mean midline shift on imaging of 6.9 mm (*SD* ± 5.3). Among CSDH patients, the median follow-up time was 57 months (range 0–213 months). A total of 528 patients (40%) died with a median time from CSDH diagnosis until death of 34 months (range 0–180). Of the 5228 matched controls, 1730 (33%) persons died during the follow-up, with a median survival time of 61 months (range 0–213). For presentation, we rounded the median survival time of CSDH patients to 36 months, i.e., 3 years.

**Table 1:** Baseline data of 1307 CSDH patients, together with comparison of survivors and non-survivors at 36 months after diagnosis

Variable	All CSDH patients (*n* = 1307)	36 months survivors vs. deceased		*p-*value
		Survivor (*n* = 1026)	Deceased (*n* = 281)	
Age (mean ± *SD*)	73.7 ± 11.4	72.0 ± 11.3	79.9 ± 9.4	< 0.001
Male sex*n*(%)	958 (73.3)	757 (73.8)	201 (71.5)	0.450
MGS on admission (mean ±*SD*)	1.76 ± 0.8	1.7 ± 0.79	1.9 ± 0.85	< 0.001
GCS on admission (mean ±*SD*)	14.19 ± 1.5	14.3 ± 1.3	13.7 ± 2.0	< 0.001
CCI score on admission (mean ±*SD*)	4.0 ± 1.9	3.65 ± 1.7	5.47 ± 1.9	< 0.001
Received surgical treatment*n*(%)	1029 (78.7)	838 (81.6)	191 (67.9)	< 0.001
Side of hematoma				
Unilateral Bilateral	955 (73.1) 352 (26.9)	752 (73.3) 274 (26.7)	203 (72.2) 78 (27.8)	0.725
Midlineshift, mm. n (mean ± SD)	6.9 ± 5.3	7.16 ± 5.2	5.89 ± 5.4	< 0.001
Frailty indicators				
Cognitive problems,*n*(%)	400 (30.6)	303 (29.5)	97 (34.5)	0.091
Frequent falling,*n*(%)	162 (12.4)	100 (9.7)	62 (22.0)	< 0.001
Unable to live independently,*n*(%)	172 (13.6)	93 (9.0)	79 (28.1)	< 0.001
Unable to perform daily self-care, *n*(%)	280 (21.4)	172 (16.7)	108 (38.4)	< 0.001
Use of benzodiazepines or psychotropic drugs,*n*(%)	226 (17.3)	150 (14.6)	76 (27.0)	< 0.001
Number of medications used *n*(mean ± *SD*)	4.8 ± 3.7	4.3 ± 3.5	6.5 ± 3.9	< 0.001

### CSDH patients; survivors vs. deceased

CSDH patients who died within 36 months after diagnosis were significantly older (79.9 ± 9.4 vs. 72.0 ± 11.3 years, *p*-value < 0.001), had higher mean MGS and CCI scores (1.9 ± 0.85 vs. 1.7 ± 0.79 and 5.47 ± 1.9 vs. 3.65 ± 1.7, both *p*-values < 0.001) and lower mean GCS score on admission (13.7 ± 2.0 vs. 14.3 ± 1.3, *p*-value < 0.001) compared to those alive at 36 months. Also, deceased patients less often received surgical treatment (838, 68% vs. 191, 81%) and had lower mean midline shift (5.89 mm. ± 5.4 vs. 7.2 mm. ± 5.2, *p*-value < 0.001).

Deceased patients more often had a history of frequent falling (62 (22%) vs. 100, (9, 7%). *p*-value < 0.001) and were more often unable to live independent or perform daily self-care (79 (28%) vs. 93 (9.0%) and 108 (38%) vs. 172 (17%), both *p*-values < 0.001)*.* Furthermore, deceased patients more often used benzodiazepines or psychotropic drugs (76 (27%) vs. 150 (15%). *p*-value < 0.001) and had a higher mean number of prescribed medications (6.5 ± 3.9 vs. 4.3 ± 3.5, *p*-value < 0.001) than surviving CSDH patients.

### Mortality and causes of death compared with matched controls

**Fig. 1:**
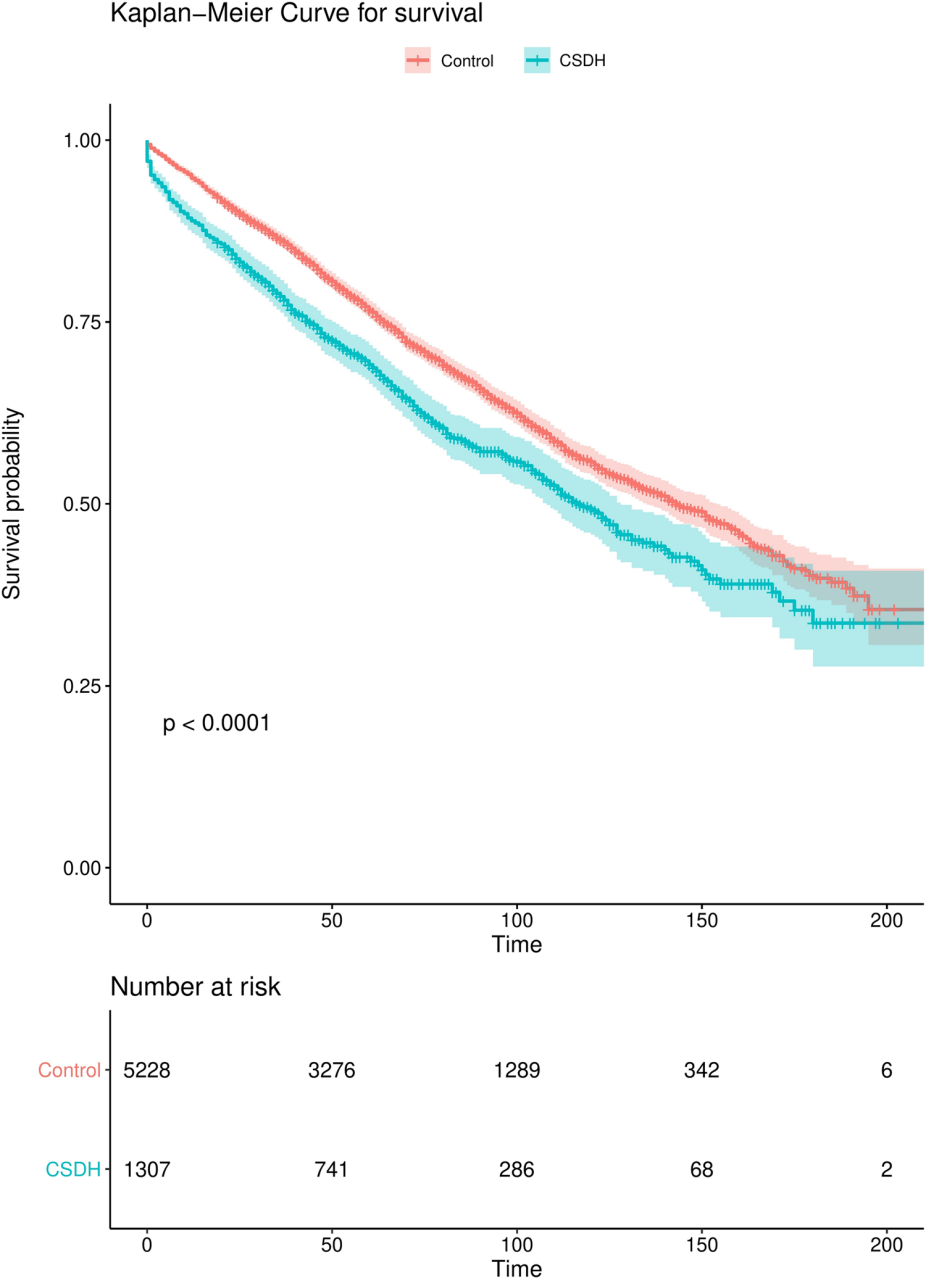
Kaplan–Meier curve for survival time in months of CSDH patients (green) vs. matched controls (red). Time on the X-axis represented in months, transparent zones around line represent 95% confidence intervals

Median duration of follow-up in all patients (CDSH patients and controls) was 56 months (range 0–213). Mortality was higher in CDSH patients compared to controls hazard ratio (HR) 1.34 (95% CI 1.2–1.5, log-rank test: < 0.0001) (Fig. 1).

Causes of death could be retrieved for 1651 (73%) of the 2258 deceased patients and controls. CSDH patients more often died of cardiovascular diseases (153 (37%) vs. 371 (30%), *p*-value < 0.001) compared to controls. Also “Accidents” which mainly consists of falls was significantly more frequent than the cause of death in CSDH patients (30 (7.2%) vs. 46 (3.7%), *p*-value 0.001) (Table 2)*.* Other causes of death did not differ between the CSDH patients and matched controls.

**Table 2:** Comparison of causes of death of CSDH patients and matched control based on data of the CBS and ICD-10 coding. The group ‘Other’ consists of hematological diseases, congenital conditions, skin diseases, bone and musculature diseases, and unspecified

Cause of death	CSDH *n* = 419	Matched controls *n* = 1232	*p*-value
Cardiovascular, including stroke, *n* (%)	153 (37)	371 (30)	< 0.001
Neoplasms, *n* (%)	92 (22)	326 (26)	0.462
Respiratory, *n* (%)	35 (8.3)	142 (12)	0.237
Psychiatric/psychic (includes some forms of dementia), *n* (%)	32 (7.6)	103 (8.3)	0.928
Accidents (traumatic events, injuries, falling), *n* (%)	30 (7.2)	46 (3.7)	0.001
Neurological (includes neurodegenerative), *n* (%)	13 (3.1)	70 (5.6)	0.090
Gastrointestinal, *n* (%)	13 (3.1)	28 (2.2)	0.204
Kidneys and urogenital system, *n* (%)	12 (2.9)	38 (3.0)	0.917
Endocrine system, *n* (%)	11 (2.6)	33 (2.6)	0.798
Infectious, *n* (%)	10 (2.3)	17 (1.3)	0.092
Other, *n* (%)	18 (4.2)	58 (4.7)	0.419

### Frailty indicators and survival

**Fig. 2:**
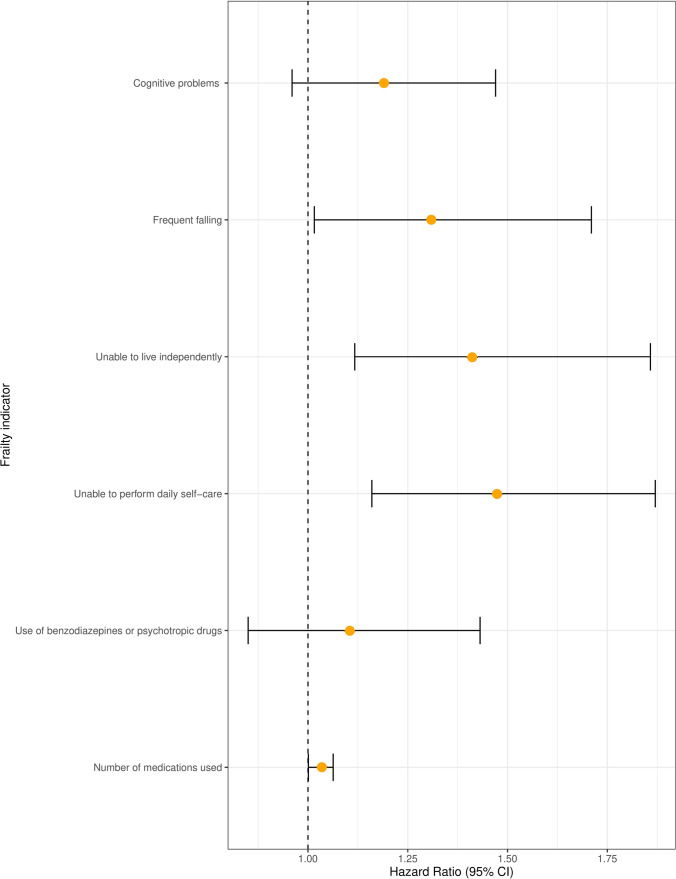
Forest plot of the association of frailty indicators and survival in a full model. Adjusted for age, sex, MGS score, CCI score, and receiving surgical treatment

Within the cohort of CSDH patients, all six frailty indicators (presence of cognitive problems, frequent falling, unable to live independently, unable to perform daily self-care, use of benzodiazepines or psychotropic drugs, and number of medications used) were associated with death. Adjustment for age, sex, receiving surgical treatment, and MGS and CCI scores on admission did not change these associations (Table 3). Combining the frailty indicators in a full model showed that frequent falling (HR 1.3 95% CI: 1.0–1.7), unable to live independently (HR 1.4 95% CI: 1.1–1.8), unable to perform daily self-care (HR 1.5 95% CI: 1.1–1.9) and number of medications used (HR 1.0 95% CI 1.00–1.1) were independently associated with survival (Fig. 2). In multivariate analysis, older age at diagnosis (*HR* = 1.05 per year (95% CI: 1.03–1.07) and higher CCI scores (1.3 per extra point) (95% CI: 1.2–1.4) independently associated with lower survival while receiving surgery lowered the chance of mortality (HR 0.48 (95% CI: 0.32–0.72). MGS score at admission and midline shift were not independently associated with survival.

**Table 3:** Hazard ratios for different frailty indicators and survival in the cohort of CSDH patients. Rounded at one decimal

Frailty indicators	Hazard ratio (95% CI) Unadjusted	Hazard ratio (95% CI) Adjusted*	Hazard ratio (95% CI) Full model**
Cognitive problems	1.2 (1.0–1.5)	1.3 (1.1–1.5)	1.2 (0.9–1.5)
Frequent falling	2.3 (1.8–2.9)	1.4 (1.1–1.8)	1.3 (1.0–1.7)
Unable to live independently	3.4 (2.7–4.2)	1.7 (1.3–2.1)	1.4 (1.1–1.8)
Unable to perform daily self-care	2.3 (1.9–2.8)	1.6 (1.3–1.9)	1.5 (1.1–1.9)
Use of benzodiazepines or psychotropic drugs	1.8 (1.5–2.2)	1.4 (1.1–1.7)	1.1 (0.9–1.4)
Number of medications used	1.1 (1.1–1.2)	1.0 (1.0–1.1)	1.0 (1.0–1.1)

^*^Adjusted for age, sex, MGS score, CCI score, and receiving surgical treatment. **Full model represents statistical analyses incorporating all frailty indicators, adjusted for age, sex, MGS score, CCI score, and receiving surgical treatment

## Discussion

We found that CSDH patients had a substantially higher long-term mortality rate than age and sex-matched controls. Furthermore, CSDH patients were more likely to die as a result of cardiovascular disease and accidents, such as falling, compared to the general population. Our hypothesis that the excess mortality of CSDH patients is related to frailty is supported by the association of several frailty indicators with mortality.

Excess mortality of CSDH patients has been reported before [12, 16, 22, 25, 29]. Mortality rates in previous studies varied from 9 to 26% at 6 months after diagnosis to 13–32% at 12 months after diagnosis. These differences in mortality could be explained by mean age of included patients and variation in follow-up time. Compared to previously reported percentages, our mortality rate at 36 months (21%) was relatively low [12, 25]. This might indicate a normalization of the excess mortality after a certain time period [25]. This is supported by the findings in Fig. 1 where mortality rates of CSDH patients and controls show large differences early in the follow-up but are almost parallel later in follow-up.

Most previous reported studies focused on specific neurological or neurosurgical aspects that influence survival, such as the use of drains, the recurrence of CSDH, or the GCS score at discharge [16, 30]. Therefore the added value of our study is that we hypothesized that underlying frailty of CSDH patients is associated with higher rates of mortality.

One argument that supports our hypothesis of frailty being related to survival in CSDH patients is the cause of death. Both cardiovascular disease and accidents were more often the cause of death in our CSDH patients than in the controls. Several studies have related cardiovascular disease to frailty with a hypothesis of age-related inflammation, sometimes dubbed “inflammageing” [14, 38]. It could also be that CSDH and cardiovascular diseases share some of the same risk factors for development such as advanced age and sex.

The majority of the accidents as cause of death consisted of some form of falling, e.g., falling out of bed or a traffic-related fall. Falling is perhaps one of the clearest outings of frailty as it depicts the deterioration in multiple domains such as the musculoskeletal and neurological systems [26, 39].

When comparing deceased vs. surviving CSDH patients, we found that deceased patients less often received surgery. This might be the result of clinical decisions for severely affected patients in which a palliative (no surgical, maximum comfort) treatment modality is chosen. More surprisingly, our univariate analysis showed that deceased patients had significantly less midline shift compared to surviving patients. However, in multivariate analysis after correcting for confounders, the amount of midline shift was not significantly associated with survival. This might be explained by the adjustment for receiving surgery, as the amount of midline shift is taken into account when deciding to perform surgery or not [36].

When combining all frailty indicators in statistical analyses (“full model”), unable to live independently, unable to perform daily self-care, frequent falling, and number of medications used were still related to mortality in our cohort of CSDH patients. Cognitive problems and the use of benzodiazepines or psychotropic drugs were not significant attributors in our full model. This might be because they are incorporated in the other tested aspects, such as number of medications used, risk of falling (for benzodiazepines or psychotropic drugs), the ability to live independently and the ability to perform daily self-care (for cognitive problems).

Recognizing frailty as a cause for mortality in CSDH patients is important as interventions to reduce frailty have been suggested to decrease mortality [31]. Some studies even report this decrease in mortality merely as the result of attention for frailty or minimal changes in hospital logistics, without the need for additional training programs or resources [17, 34]. Specific programs for improvement have focused on nutritional status, physical components such as gait speed and muscle strength, or social support [11]. There are also recommendations for perioperative management of frailty that could be beneficial for our CSDH patients. The most often suggested improvement in perioperative frailty is to increase attention for frailty by implementing frailty screening tools [1, 2]. While this seems to be obvious, the implementation of orthogeriatrics, an intensive collaboration between Geriatrics and Orthopedic Surgery, focusing on the optimal perioperative care for elderly patients with (fall-related) fractures, has shown to lead to better survival after hip fractures [18, 24]. Whether the implementation of a similar “neurogeriatrics” department could be equally effective in reducing CSDH mortality requires more study on the role of frailty in CSDH. We suggest to a prospective study that determines frailty using a comprehensive frailty measurement tool combined with a long follow-up time in both a CDSH patient and control group.

### Strengths and limitations

A strength of our study is that it comprises a large sample of CSDH patients with a solid outcome measure (survival) from a high-quality and complete data source (Dutch national statistics). Also, this is the first study to investigate the causes of the reported excess mortality of CSDH patients.

Our study has several limitations. Ideally, we would have measured frailty with an extensive frailty scale. However, the most comprehensive frailty scales require a form of face-to-face contact to establish certain physical or cognitive components (nutritional status, gait speed, weight loss, ability to stand up) which was not possible in our retrospective cohort. We do believe that our frailty indicators provide a good insight into the frailty status of our patients, nevertheless reporting bias may have occurred. For instance, frequent falling or presence of cognitive problems may not have been actively asked and noted by the treating physician. In one of the participating centers, healthy aging is core research theme which might have resulted in improved attention for frail patients. In addition, it is quite common for CSDH patients to receive medicinal treatment with dexamethasone in the Netherlands. In our cohort, 21% of the patients received dexamethasone (alone or in combination with surgery), whereas more recent insights show that dexamethasone might be related to worse outcome. However, since the potential negative effect of dexamethasone is relatively small and it concerns only a fifth of our population, we do not expect our results to be affected. Therefore, we consider the potential impact of these differences small and believe that our conclusions are generalizable to other Western countries.

## Conclusion

CSDH patients have higher mortality rates than the general population. CSDH patients more often die as a result from cardiovascular diseases and falls. Mortality in CSDH patients is associated with frailty indicators, making prospective studies on frailty in CSDH patients and mortality warranted.

## Data Availability

The data that support the findings of this study are available from the corresponding author, upon reasonable request and in compliance with the medical-ethical and privacy regulations.
